# Supplementary figures and images for: Humans adapt rationally to approximate estimates of uncertainty
Source: eLife. 2025 Jul 8;14:RP103734. doi: 10.7554/eLife.103734 (PMC12237418; doi:10.7554/eLife.103734)

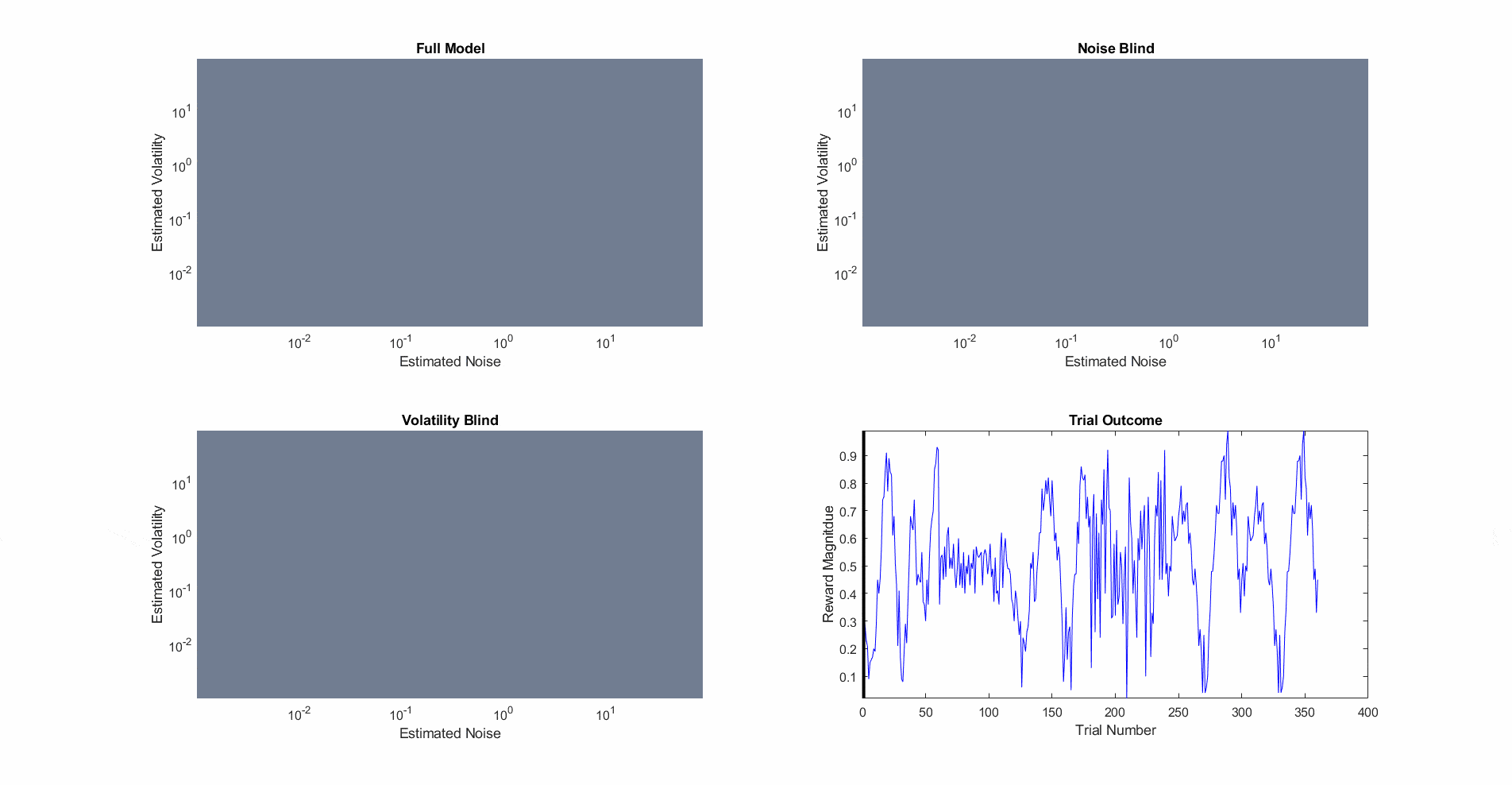

Supplement: Supplementary file 1 [file elife-103734-animation1.gif]
